# 3D-QSAR/CoMFA-Based Structure-Affinity/Selectivity Relationships of Aminoalkylindoles in the Cannabinoid CB1 and CB2 Receptors

**DOI:** 10.3390/molecules19032842

**Published:** 2014-03-05

**Authors:** Jaime Mella-Raipán, Santiago Hernández-Pino, César Morales-Verdejo, David Pessoa-Mahana

**Affiliations:** 1 Departamento de Química y Bioquímica, Facultad de Ciencias, Universidad de Valparaíso, Av. Gran Bretaña 1111, Playa Ancha, Valparaíso, Casilla 5030, Chile; 2 Laboratorio de Bionanotecnología, Departamento de Ciencias Químico Biológicas, Universidad Bernardo O´Higgins, General Gana 1780, Santiago 8370993, Chile; 3 Departamento de Farmacia, Facultad de Química, Pontificia Universidad Católica de Chile, Casilla 306, 22, Santiago 7820436, Chile

**Keywords:** cannabinoid, CB1/CB2, aminoalkylindole, molecular modelling, 3D-QSAR, Comparative Molecular Field Analysis (CoMFA), simulated annealing, structure-activity relationships

## Abstract

A 3D-QSAR (CoMFA) study was performed in an extensive series of aminoalkylindoles derivatives with affinity for the cannabinoid receptors CB1 and CB2. The aim of the present work was to obtain structure-activity relationships of the aminoalkylindole family in order to explain the affinity and selectivity of the molecules for these receptors. Major differences in both, steric and electrostatic fields were found in the CB1 and CB2 CoMFA models. The steric field accounts for the principal contribution to biological activity. These results provide a foundation for the future development of new heterocyclic compounds with high affinity and selectivity for the cannabinoid receptors with applications in several pathological conditions such as pain treatment, cancer, obesity and immune disorders, among others.

## 1. Introduction

The cannabinoid receptors are seven transmembrane domains proteins with two known subtypes: CB1 and CB2 [1]. They belong to the G protein-coupled receptors (GPCRs) and since the discovery of ∆9-THC [2], the main psychoactive component of *Cannabis sativa*, they have been the focus of several studies due to their implication in a variety of pathophysiological conditions [3,4]. There is a notable difference in the distribution of the cannabinoid receptors, as well as in the physiological functions they control [5]. However, there are some regions that can express both subtypes of receptors [6]. The CB1 receptors are expressed fundamentally in the central nervous system (CNS) and they are the most abundant GPCRs in the brain, with levels 10-fold higher than those of other GPCRs [7], indicating a highly significant functional role in a wide variety of circuits and neuronal systems [8]. The greater abundance of CB1 receptors in the CNS occurs in areas related to the control of motor activity, such as cortex, basal ganglia and cerebellum [9,10,11]. At a peripheral level the CB1 receptors are located in organs such as testes, vas deferens, bladder, ileum, eyes, liver, skeletal muscle, heart, pancreas and adipose tissue [12]. It is postulated that both central and peripheral distribution of CB1 receptors are integrated in the regulation of metabolic homeostasis [13], so compounds that target these receptors can be useful in the treatment of obesity, type 2 diabetes and other metabolic disorders. On the other hand, the distribution of the CB2 receptors is more bounded, as they are found almost exclusively in cells of the immune system, with particularly high levels in B lymphocytes and natural killer cells [14,15]. Other sites where CB2 receptors are found include thymus, tonsils, bone marrow, spleen, pancreas, peripheral nerve terminals, microglial cells, tumor cells (melanoma and glioma) and astrocytes [16,17,18,19]. Little is known about the physiological and pathophysiological role of CB2 receptors. When activated, they can modulate the migration of immune system cells, suppress the release of proinflammatory cytokines and increase the release of inflammatory cytokines [20,21]. Although the physiological role of CB2 receptors is still not completely understood, several preclinical studies support the utility of using CB2 ligands for the treatment of chronic pain, maintenance of bone density, halting the progression of atherosclerotic lesions, asthma, autoimmune and inflammatory diseases, and multiple sclerosis [22].

**Figure 1:**
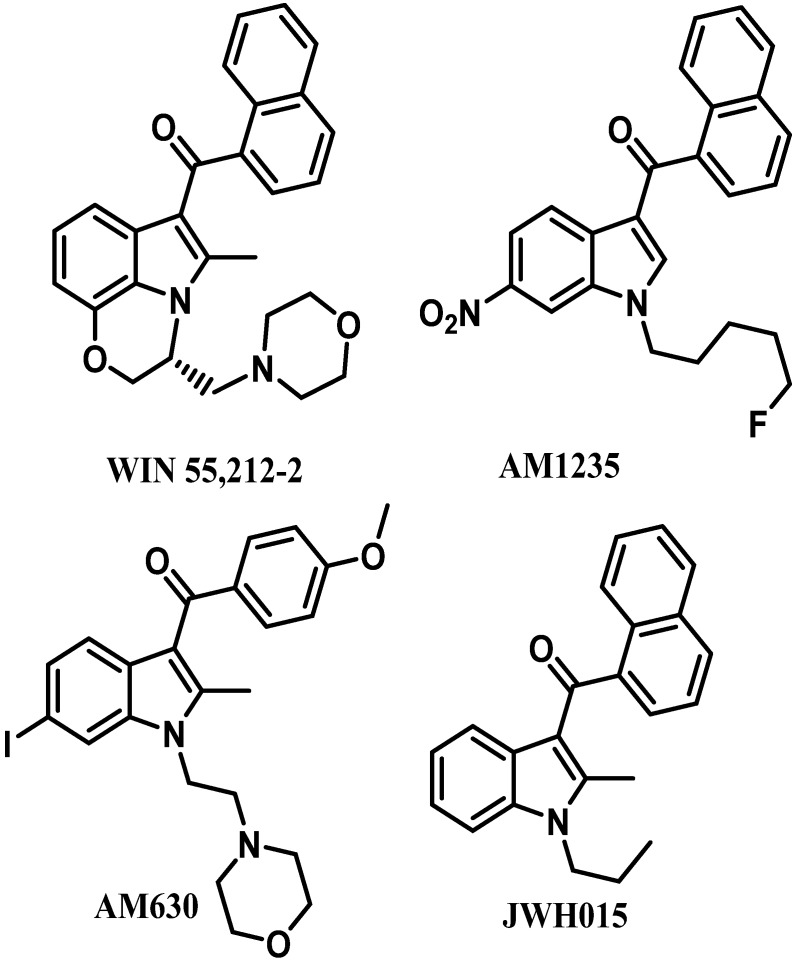
Representative aminoalkylindole ligands.

Among the diverse variety of compounds synthesized against the CB receptors, the aminoalkylindole family represents a versatile group of compounds that includes CB1 and CB2 ligands such as WIN55212, AM1235, AM630 and JWH-015 (Figure 1). However, despite their chemical similarity, there is no obvious chemical rationality to the distinct affinity they exhibit for cannabinoid receptors, so in order to obtain useful information about the three-dimensional requirements for their receptor selectivity, we have performed a CoMFA study on a wide range of selective CB2 aminoalkylindoles reported in recent literature [23,24,25]. Moreover, the information obtained in this study will provide a means for predicting the activity of related compounds, and help guide further structural modifications and synthesis of new potent and selective cannabinoid ligands.

From the work of Cramer *et al.* [26], CoMFA represent a useful methodology in understanding the pharmacological properties of a studied series of compounds. The steric and electrostatic maps obtained may help to: (a) understand the nature of the ligand-receptor interactions; (b) predict the biological affinity and (c) rationally design new promising compounds. Some Three-dimensional Quantitative Structure-Activity Relationships (3D-QSAR) studies have been made on the cannabinoid ligands for the CB1 or CB2 receptors [27,28,29,30,31] in the last years. As a continuation of our efforts aimed at exploring and understanding the cannabinoid system [32,33,34], and due the need to obtain useful SAR in the aminoalkylindole family, we present two CoMFA models carried out on a wide number of compounds of the recent literature [23,24,25] with a marked pKi ratio (CB1/CB2). The molecules have broad structural variability and the contour plots are vivid and clear, and offer valuable information about the structural requirements for cannabinoid affinity and selectivity.

## 2. Results and Discussion

### 2.1. Cannabinoid CB1 and CB2 CoMFA Models

3D-QSAR models were obtained from CoMFA analysis and its statistical parameters are listed in Table 1. For a reliable predictive model the square q^2^ of the cross-validation coefficient should be greater than 0.5 [35]. The models have high r^2^, r^2^_pred_ and q^2^ suggesting that they are reliable and predictive. The steric and electrostatic contributions were found to be 71% and 29% respectively, in both cases, which is in agreement with the hydrophobic character of the cannabinoid ligands and the active site of the receptors. 

**Table 1:** Statistical parameters of the CoMFA models ^a^.

CoMFA CB1								%Contribution	
N	q^2^	r^2^	SEE	F	PRESS	SD	r^2^_pred_	Steric	Electrostatic
2	0.722	0.845	0.197	100	5.46	15.22	0.641	71.4	28.6
**CoMFA CB2**									
10	0.643	0.999	0.021	4376	2.56	10.16	0.748	71.2	28.8

^a^ N is the optimum number of components, q^2^ is the square of the LOO cross-validation (CV) coefficient, r^2^ is the square of the non CV coefficient, r^2^_pred_ is the predictive r^2^ based only on the test set molecules, SEE is the standard error of estimation of non CV analysis, SD is the sum of the squared deviation between the biological activity of molecules in the test set and the mean activity of the training set molecules, PRESS is the sum of the squared deviations between predicted and actual biological activity values for every molecule in the test set, and F is the F-test value.

The affinity values of molecules predicted by CoMFA models are listed in Table 2. In the CB1 model, the compounds have a residual range of ‒0.48 to 0.95 (including training and test set). While in the CB2 model, the compounds have a residual of ‒0.63 to 0.65 (including training and test set). The major deviations from activity were in compounds **38** and **39** in CB1 and **84** and **88** in CB2.

**Table 2:** Experimental and predicted activity for training and test set in the CB1 and CB2 models ^a^.

		CoMFA CB1		
Molecule	K_i_ CB1	Actual pK_i_	Predicted pK_i_	Residual
	(nM)	(M)	(M)	
**Training Set**				
**1**	1660	5.78	5.664	0.12
**2**	488	6.312	6.223	0.09
**3**	698	6.156	6.325	−0.17
**4**	530	6.276	6.352	−0.08
**5**	3617	5.442	5.462	−0.02
**6**	2791	5.554	5.526	0.03
**7**	3621	5.441	5.531	−0.09
**8**	1258.9	5.9	5.918	−0.02
**9**	389	6.41	6.353	0.06
**10**	245.5	6.61	7.022	−0.41
**11**	1096.5	5.96	5.958	0.00
**12**	281.8	6.55	6.216	0.33
**13**	2818.4	5.55	5.665	−0.12
**14**	776.2	6.11	5.978	0.13
**15**	851.1	6.07	6.065	0.01
**16**	1288.2	5.89	5.911	−0.02
**17**	1698.2	5.77	5.775	−0.01
**18**	1862.1	5.73	5.808	−0.08
**19**	229.1	6.64	6.417	0.22
**20**	363.1	6.44	6.551	−0.11
**21**	616.6	6.21	6.07	0.14
**22**	131.8	6.88	6.907	−0.03
**23**	61.7	7.21	7.036	0.17
**24**	72.4	7.14	6.948	0.19
**25**	537	6.27	6.176	0.09
**26**	562.3	6.25	6.13	0.12
**27**	91.2	7.04	6.859	0.18
**28**	213.8	6.67	6.695	−0.03
**29**	281.8	6.55	6.921	−0.37
**30**	144.5	6.84	6.605	0.24
**31**	676.1	6.17	6.083	0.09
**32**	660.7	6.18	6.222	−0.04
**33**	380.2	6.42	6.723	−0.30
**34**	213.8	6.67	6.635	0.04
**35**	691.8	6.16	6.487	−0.33
**36**	1258.9	5.9	6.12	−0.22
**Test Set**				
**37**	1828.1	5.738	6.141	−0.4
**38**	12.3	7.91	6.999	0.91
**39**	13.2	7.88	6.93	0.95
**40**	44.7	7.35	6.886	0.46
**41**	33.1	7.48	6.73	0.75
**42**	28.2	7.55	6.762	0.79
**43**	1000	6	6.462	−0.46
**44**	31.6	7.5	6.773	0.73
**45**	25.1	7.6	6.825	0.77
**46**	1621.8	5.79	6.218	−0.43
**47**	354.8	6.45	6.927	−0.48
**48**	47.9	7.32	6.883	0.44
**49**	2238.7	5.65	6.124	−0.47
**Training Set**				
**1**	110	6.959	6.974	−0.02
**2**	98	7.009	7.028	−0.02
**3**	67	7.174	7.169	0.01
**4**	50	7.301	7.33	−0.03
**5**	10.3	7.987	7.964	0.02
**7**	6.4	8.196	8.174	0.02
**10**	3.2	8.495	8.536	−0.04
**11**	3.3	8.481	8.478	0.00
**12**	4.6	8.337	8.348	−0.01
**15**	4.4	8.357	8.385	−0.03
**17**	26	7.585	7.522	0.06
**20**	11	7.959	7.972	−0.01
**21**	16	7.796	7.811	−0.02
**23**	2.6	8.585	8.579	0.01
**24**	2.1	8.678	8.647	0.03
**25**	5.9	8.229	8.234	−0.01
**27**	2.8	8.553	8.545	0.01
**28**	3.3	8.481	8.5	−0.02
**30**	1.8	8.745	8.73	0.02
**34**	8.2	8.086	8.079	0.01
**35**	2.8	8.553	8.542	0.01
**37**	17.8	7.75	7.743	0.01
**41**	0.9	9.056	9.06	0.00
**43**	2.9	8.538	8.521	0.02
**45**	1.4	8.854	8.847	0.01
**49**	31	7.509	7.489	0.02
**50**	50	7.301	7.299	0.00
**51**	189	6.724	6.719	0.01
**52**	12.7	7.896	7.907	−0.01
**53**	25.4	7.595	7.567	0.03
**54**	11.2	7.951	7.98	-0.03
**55**	22.2	7.654	7.651	0.00
**56**	11.6	7.936	7.964	−0.03
**57**	2.5	8.606	8.608	0.00
**58**	2	8.693	8.691	0.00
**59**	70.8	7.15	7.134	0.02
**60**	5	8.301	8.299	0.00
**61**	1.6	8.796	8.806	−0.01
**62**	3	8.523	8.498	0.03
**63**	2.5	8.602	8.635	−0.03
**64**	3.5	8.456	8.452	0.00
**65**	8.5	8.071	8.082	−0.01
**66**	0.5	9.292	9.273	0.02
**67**	9.3	8.032	8.028	0.00
**68**	3.1	8.509	8.504	0.01
**69**	9.3	8.032	8.039	−0.01
**70**	27	7.569	7.556	0.01
**71**	32	7.495	7.502	−0.01
**72**	1.9	8.721	8.743	−0.02
**73**	5.8	8.237	8.254	−0.02
**74**	9.2	8.036	8.01	0.03
**75**	2	8.699	8.671	0.03
**76**	2.1	8.678	8.661	0.02
**77**	2.2	8.658	8.644	0.01
**78**	3.1	8.509	8.532	−0.02
**79**	2.9	8.538	8.554	−0.02
**80**	20	7.699	7.721	−0.02
**81**	8.3	8.081	8.066	0.02
**82**	1.3	8.886	8.884	0.00
**83**	35	7.456	7.455	0.00
**Test Set**				
**6**	5.7	8.242	8.202	0.04
**8**	3	8.523	8.553	−0.03
**9**	3.9	8.409	8.651	−0.24
**18**	58	7.237	7.164	0.07
**26**	2.3	8.638	8.205	0.43
**32**	1.3	8.886	8.494	0.39
**33**	1	9.004	8.606	0.40
**36**	11.5	7.939	8.209	−0.27
**39**	0.7	9.187	8.635	0.55
**42**	1	9	8.53	0.47
**48**	3.7	8.432	8.264	0.17
**84**	11.8	7.928	7.283	0.65
**85**	20.5	7.688	7.353	0.34
**86**	70	7.155	7.346	−0.19
**87**	6.6	8.18	8.353	-0.17
**88**	190	6.721	7.35	−0.63
**89**	3.8	8.42	8.662	−0.24
**90**	0.4	9.398	8.863	0.53
**91**	1.8	8.745	8.715	0.03
**92**	1.8	8.745	8.557	0.19

^a^ The structures of all the molecules are displayed on Table 3.

**Figure 2:**
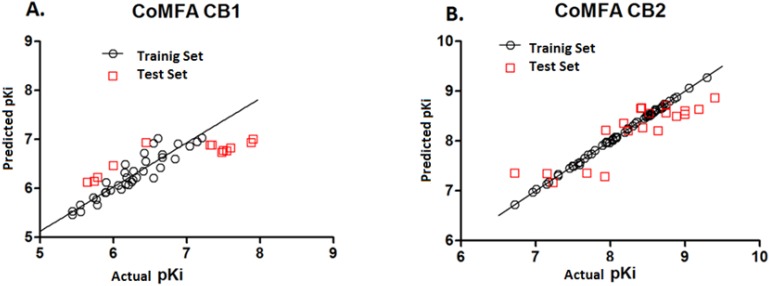
Experimental *versus* predicted activity for **A**. CB1 CoMFA analysis and **B**. CB2 CoMFA analysis.

The plot of the predicted pKi values *versus* the experimental ones for CoMFA analysis is also shown in Figure 2, in which most points are well distributed along the line Y = X, suggesting that the quality of the 3D-QSAR model is good.

### 2.2. CoMFA Contour Maps Analysis

**Figure 3:**
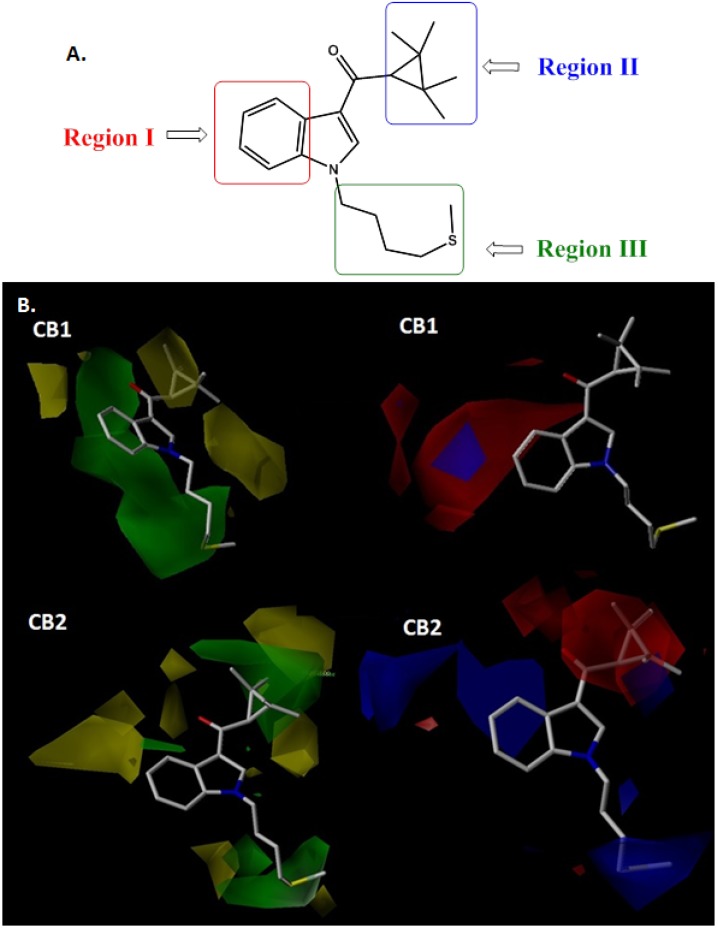
**A**. Molecular Regions analyzed. **B**. CoMFA steric (left) and electrostatic (right) contour maps for CB1 and CB2 receptors around compound **90**, the most active of the series. Sterically favored (green) and disfavored (yellow); electropositive (blue) and electronegative (red).

CoMFA contour maps analysis was performed to visualize the important region in 3D molecules where the steric and electrostatic fields may affect the affinity and selectivity of the studied compounds in the cannabinoid CB1 and CB2 receptors. The weight of StDev*Coeff was used to calculate the field energies for all fields in CoMFA models. The highly active compound **90** was shown as the template ligand for all contour maps. CB1 and CB2 contour maps were generated from binding affinity of series of indole ligands evaluated at recombinant human CB1 and CB2 receptors. The steric and electrostatic contour maps of CoMFA are displayed in Figure 3. In order to systematize the analysis and obtain useful information on SAR, we had divided the molecule into three regions:

*Region I*. Close examination of the CoMFA maps allows us to see in the steric CB1 model a large green polyhedron around this region, which continues along the entire region III. At a greater distance from the green polyhedron in region I, we can see a yellow region. This suggests that bulky substituents at any position of the benzene will increase biological activity and selectivity for this receptor. However, as it was already mentioned, the yellow region restricts the size and length of the substitutions to some extent in the position 5 of the indole system. In the steric CB2 model, the big restrictive contour map near the positions 4 and 5 of the indole core limits drastically the possibility of substitute these positions. In fact, the most active compounds in the CB2 receptor (compounds **33**, **39**, **41**, **66** and **90**) bear a hydrogen atom in positions 4 and 5. On the other hand, in the electrostatic contour maps, the CB1 model indicates a red area contiguous to the positions 4 and 5 of the indole nucleus. According to this, electronegative substituents can be connected to the benzene or it is possible to replace the ring by an isostere with electronegative properties such as a thiophene or pyrrole. In the CB2 model the data implicate an inverse situation. In this case would be favorable the insertion of electropositive groups or the exchange for rings capable of protonation at physiological pH, such as pyridine, pyrimidine, piperidine and aniline, among others. Region I is, therefore, from the electronic standpoint a key area for selectivity.

*Region II*. The steric contour maps show that region II is inside a yellow polyhedron in CB1, while it is inside a green contour map surrounded by two yellow areas in CB2. This noteworthy difference suggests that a controlled increase of the size of the ring substituents or the replacement of this ring for bulkier systems like naphthoyl (for example in compounds **82** and **83**), will raise the selectivity for the CB2 receptor. In fact, the comparative lower CB2 receptor Ki values for compounds **38**–**42** (Ki < 20 nM), are consistent with the existence of bulky rings in region II, and, as expected, they displayed less affinity for the CB1 receptor (Ki > 60 nM in all cases). On the other hand, there are not electrostatic surfaces around the region II in the CB1 model. The absence of information in this particular region is consequence of the similarity of the rings occupying that area which are electrically neutral: cyclopropyl, adamantyl, cyclohexyl, and phenyl. This, however, rather than being a limitation in the design, provides the ability to evaluate multiple electronic options. However, the CoMFA for CB2 shows the region II completely immersed in a red polyhedron. This suggests that when adding electronegative atoms or groups such as oxygen, halogens, sulfur, and no protonable nitrogens in this region, the CB2 affinity is boosted. As an example, compound **25**, bearing an oxaadamantyl moiety in this region, and compound **36**, with a 2-iodo-5-nitrophenyl, have 90 and 110 times greater affinity for the CB2 receptor than CB1, respectively.

*Region III.* As can be seen for compound **90**, there is a green surface along the *N*-alkyl chain in the CB1 model but only a terminal green polyhedron in the CB2 model. This suggests that a branched chain would increase the affinity for the CB1 receptor, and the substitution with bulky groups at the end of it would be better for the CB2 affinity. Analogously to region II, in region III there are not any electrostatic contour maps in CB1 CoMFA model, while a blue region is observed in the CB2 receptor. This suggests that the introduction of protonable or electropositive groups would increase CB2 affinity. For example compounds **49**, **72**, **73** and **80** share the same structure but differ in the type of nitrogen at the end of the N-alkyl chain. Compounds **49** and **80** have the less basic nitrogen, whereas compounds **72** and **73** have strong basic sp3 nitrogen displaying 10 times higher CB2 affinity than compounds **49** and **80**. This may suggests the presence of a hydrogen bond or ionic interaction in the stabilization of the complex ligand-receptor.

## 3. Experimental

### 3.1. Data Set

A structurally diverse and homogeneous data set of 92 aminoalkylindole ligands with binding affinities (expressed as Ki) spanning about 4 log orders of magnitude (12.3–3,621 nM for CB1 and 0.4–190 nM for CB2), was selected from literature [23,24,25] for the construction of the CoMFA models (Table 3). The data set was classified into training set (36 compounds in CB1 and 60 compounds in CB2) and test set (13 compounds in CB1 and 20 compounds in CB2) in such a way to avoid any redundancy in terms of structural features or activity range and to assess the predictive ability of the models.

**Table 3:** Molecular structures of the molecules. 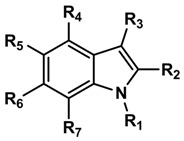

Comp.	R_1_	R_2_	R_3_	R_4_	R_5_	R_6_	R_7_
**1**	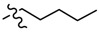	**H**	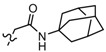	**H**	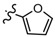	**H**	**H**
**2**	**CH_3_-**	**H**	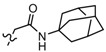	**H**	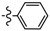	**H**	**H**
**3**	**CH_3_CH_2_-**	**H**	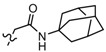	**H**	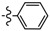	**H**	**H**
**4**	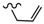	**H**	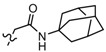	**H**	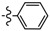	**H**	**H**
**5**	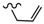	**H**	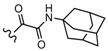	**H**	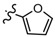	**H**	**H**
**6**	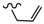	**H**	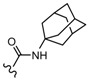	**H**	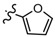	**H**	**H**
**7**	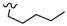	**H**	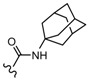	**H**	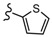	**H**	**H**
**8**	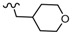	**H**	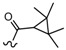	**H**	**H**	**CH_3_SO_2_-**	**H**
**9**	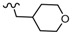	**H**	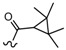	**OH-**	**H**	**H**	**H**
**10**	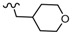	**H**	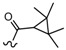	**H**	**H**	**H**	**OH-**
**11**	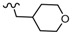	**H**	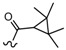	**CH_3_O-**	**H**	**H**	**H**
**12**	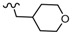	**H**	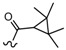	**H**	**CH_3_O-**	**H**	**H**
**13**	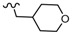	**H**	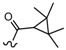	**H**	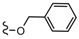	**CH_3_O-**	**H**
**14**	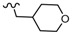	**H**	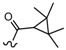	**H**	**OH-**	**CH_3_O-**	**H**
**15**	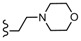	**H**	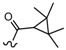	**H**	**H**	**H**	**H**
**16**	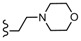	**H**	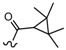	**H**	**H**	**CH_3_O-**	**H**
**17**	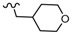	**H**	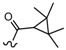	**H**	**CH_3_OCH_2_-**	**H**	**H**
**18**	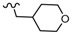	**H**	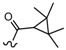	**H**	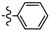	**H**	**H**
**19**	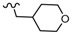	**H**	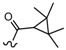	**H**	**H**	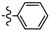	**H**
**20**	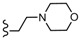	**H**	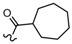	**H**	**H**	**H**	**H**
**21**	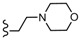	**H**	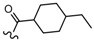	**H**	**H**	**H**	**H**
**22**	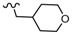	**H**	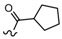	**H**	**H**	**H**	**H**
**23**	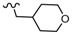	**H**	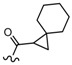	**H**	**H**	**H**	**H**
**24**	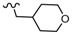	**H**	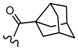	**H**	**H**	**H**	**H**
**25**	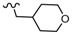	**H**	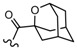	**H**	**H**	**H**	**H**
**26**	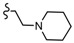	**H**	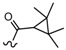	**H**	**H**	**H**	**H**
**27**	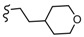	**H**	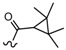	**H**	**H**	**H**	**H**
**28**	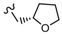	**H**	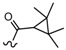	**H**	**H**	**H**	**H**
**29**	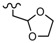	**H**	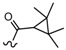	**H**	**H**	**H**	**H**
**30**	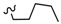	**H**	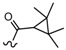	**H**	**H**	**H**	**H**
**31**	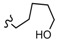	**H**	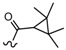	**H**	**H**	**H**	**H**
**32**	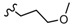	**H**	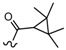	**H**	**H**	**H**	**H**
**33**	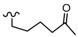	**H**	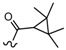	**H**	**H**	**H**	**H**
**34**	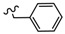	**H**	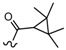	**H**	**H**	**H**	**H**
**35**	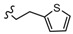	**H**	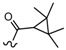	**H**	**H**	**H**	**H**
**36**	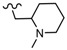	**H**	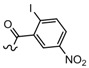	**H**	**H**	**H**	**H**
**37**	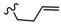	**H**	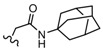	**H**	**H**	**H**	**H**
**38**	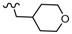	**H**	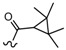	**H**	**H**	**H**	**H**
**39**	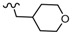	**H**	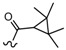	**H**	**H**	**Br-**	**H**
**40**	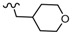	**H**	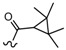	**H**	**H**	**H**	**CH_3_O-**
**41**	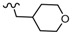	**H**	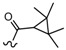	**H**	**H**	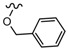	**H**
**42**	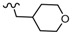	**H**	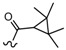	**H**	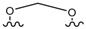		**H**
**43**	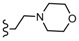	**H**	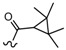	**H**	**H**	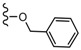	**H**
**44**	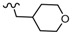	**H**	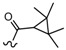	**H**	**H**	**CN-**	**H**
**45**	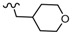	**H**	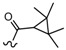	**H**	**H**	**CH_3_OCO-**	**H**
**46**	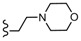	**H**	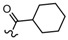	**H**	**H**	**H**	**H**
**47**	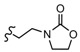	**H**	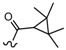	**H**	**H**	**H**	**H**
**48**	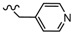	**H**	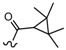	**H**	**H**	**H**	**H**
**49**	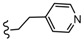	**H**	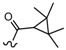	**H**	**H**	**H**	**H**
**50**	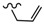	**H**	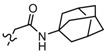	**H**	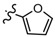	**H**	**H**
**51**	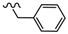	**H**	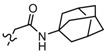	**H**	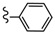	**H**	**H**
**52**	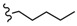	**H**	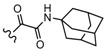	**H**	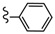	**H**	**H**
**53**	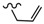	**H**	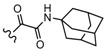	**H**	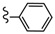	**H**	**H**
**54**	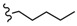	**H**	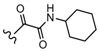	**H**	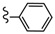	**H**	**H**
**55**	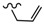	**H**	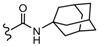	**H**	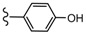	**H**	**H**
**56**	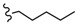	**H**	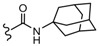	**H**	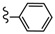		
**57**	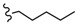	**H**	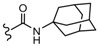	**H**	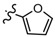	**H**	**H**
**58**	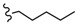	**H**	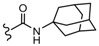	**H**	**H**	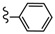	**H**
**59**	**H**	**H**	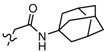	**H**	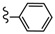	**H**	**H**
**60**	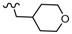	**H**	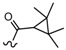	**F-**	**F-**	**F-**	**F-**
**61**	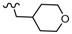	**H**	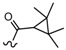	**H**	**F-**	**H**	**H**
**62**	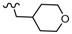	**H**	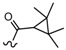	**H**	**Cl-**	**H**	**H**
**63**	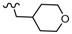	**H**	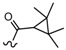	**H**	**H**	**Cl-**	**H**
**64**	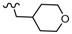	**H**	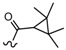	**H**	**H**	**CF_3_-**	**H**
**65**	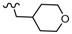	**H**	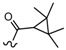	**H**	**H**	**OH-**	**H**
**66**	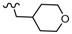	**H**	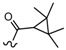	**H**	**H**	**CH_3_O-**	**H**
**67**	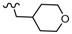	**H**	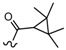	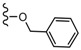	**H**	**H**	**H**
**68**	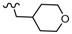	**H**	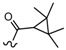	**H**	**H**	**H**	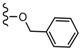
**69**	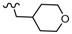	**H**	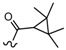	**H**	**NH_2_-**	**H**	**H**
**70**	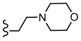	**CH_3_-**	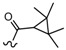	**H**	**H**	**H**	**H**
**71**	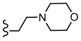	**H**	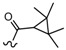	**NH_2_-**	**H**	**H**	**H**
**72**	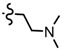	**H**	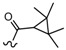	**H**	**H**	**H**	**H**
**73**	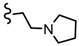	**H**	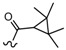	**H**	**H**	**H**	**H**
**74**	**CH_3_(CH_2_)_2_-**	**H**	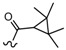	**H**	**H**	**H**	**H**
**75**	**CH_3_(CH_2_)_3_-**	**H**	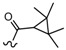	**H**	**H**	**H**	**H**
**76**	**OH(CH_2_)_3_-**	**H**	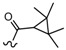	**H**	**H**	**H**	**H**
**77**	**OH(CH_2_)_4_-**	**H**	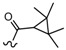	**H**	**H**	**H**	**H**
**78**	**CH_3_O(CH_2_)_2_-**	**H**	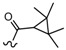	**H**	**H**	**H**	**H**
**79**	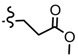	**H**	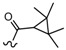	**H**	**H**	**H**	**H**
**80**	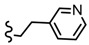	**H**	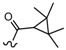	**H**	**H**	**H**	**H**
**81**	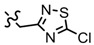	**H**	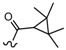	**H**	**H**	**H**	**H**
**82 ^a^**	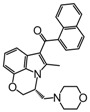						
**83 ^b^**	**CH_3_(CH_2_)_2_-**	**CH_3_-**	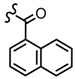	**H**	**H**	**H**	**H**
**84**	**CH_3_(CH_2_)_2_-**	**H**	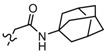	**H**	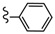	**H**	**H**
**85**	**CH_3_(CH_2_)_3_-**	**H**	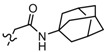	**H**	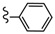	**H**	**H**
**86**	**CH_3_(CH_2_)_4_-**	**H**	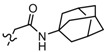	**H**	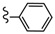	**H**	**H**
**87**	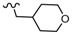	**H**	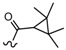	**H**	**Br-**	**H**	**H**
**88**	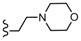	**H**	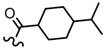	**H**	**H**	**H**	**H**
**89**	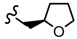	**H**	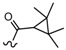	**H**	**H**	**H**	**H**
**90**	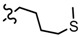	**H**	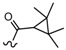	**H**	**H**	**H**	**H**
**91**	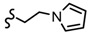	**H**	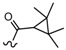	**H**	**H**	**H**	**H**
**92**	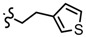	**H**	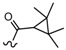	**H**	**H**	**H**	**H**

^a^ (*R*)-(+)-WIN 55,212-2; ^b^ JWH-015.

### 3.2. Generation of CoMFA and Partial Least Squares (PLS) Analysis

**Figure 4:**
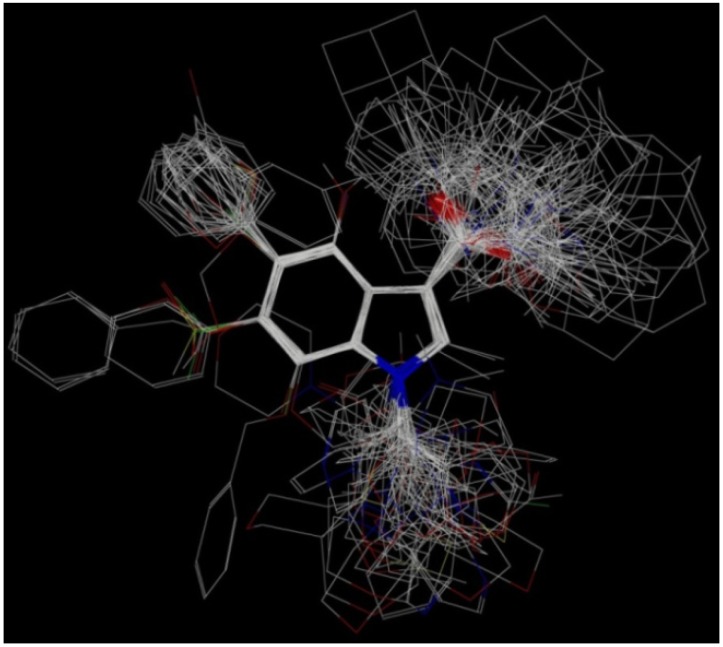
The superimposed structure of all compounds used in the CoMFA models.

CoMFA studies were performed using SYBYL-X 1.2 molecular modeling software (Tripos Inc., St. Louis, MO, USA) running on a PC platform with Intel core i7 CPU. The compounds were subjected to a preliminary minimization to remove close atom contacts by 1,000 cycles of minimization using standard Tripos force field [36] (with 0.005 kcal/mol energy gradient convergence criterion). The structures were next subjected to molecular dynamic simulation to heat the molecule at 700 K for 1,000 fs followed by annealing the molecule to 200 K for 1,000 fs. Gasteiger-Hückel charges [37] were assigned to all the molecules. Finally the minimized structures were superimposed by the atom fit method choosing the indole nucleus as the common scaffold for alignment (Figure 4).

PLS analysis was used to construct a linear correlation between the CoMFA descriptors (independent variables) and the activity values (dependent variables) [38]. To select the best model, the cross-validation analysis was performed using the LOO method (and SAMPLS), which generates the square of the cross-validation coefficient (q^2^) and the optimum number of components N. The optimum number of components analysis is shown in Table 4.The non-cross-validation was performed with a column filter value of 2.0 to speed up the analysis and reduce the noise.

**Table 4:** Optimum number of components analysis ^a^.

**CoMFA CB1**															
**SEP**	0.268	**0.263**	0.273	0.277	0.284	0.297	0.305	0.311	0.318	0.322	0.329	0.335	0.341	0.348	0.355
**q^2^**	0.704	**0.722**	0.709	0.709	0.703	0.685	0.677	0.674	0.671	0.673	0.672	0.673	0.672	0.672	0.672
**N**	1	**2**	3	4	5	6	7	8	9	10	11	12	13	14	15
**CoMFA CB2**															
**SEP**	0.432	0.392	0.388	0.374	0.373	0.369	0.366	0.370	0.374	**0.375**	0.377	0.380	0.383	0.387	0.391
**q^2^**	0.440	0.546	0.563	0.600	0.611	0.625	0.638	0.639	0.637	**0.643**	0.647	0.649	0.650	0.650	0.651
**N**	1	2	3	4	5	6	7	8	9	**10**	11	12	13	14	15

^a^ SEP = standard error of prediction; q^2^ = the square of the LOO cross-validation (CV) coefficient; N = the optimum number of components.

To assess the predictive ability of the models, the pKi values of the test sets were predicted and then was calculated the predictive r^2^ (r^2^_pred_) [39,40] for both CoMFA models. r^2^_pred_, which measures the predictive performance of a PLS model, is defined by equation 1 as follows:

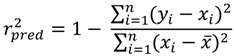
(1)
where *y_i_* is the predicted biological activity value of every molecule in the test set, *x_i_* is the actual biological activity value of every molecule in the test set, and *x* is the mean activity of the training set molecules.

## 4. Conclusions

**Figure 5:**
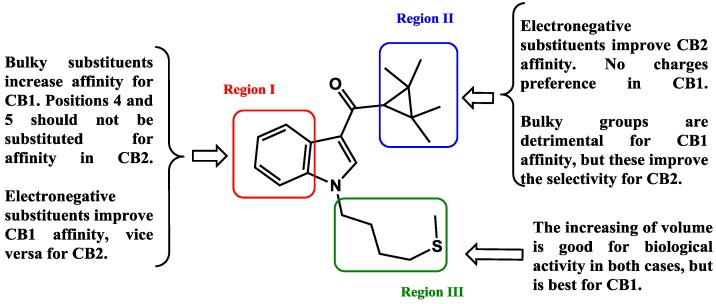
Structure-affinity/selectivity relationships derived from CoMFA studies developed in this work.

In summary, a 3D-QSAR study was performed on a wide series of 92 aminoalkylindoles with the aim of understanding and rationalizing their affinity and selectivity for the cannabinoid receptors CB1 and CB2. We have defined clear differences in the steric and electrostatic requirements for each receptor subtype. In Figure 5 we summarize the structure-activity relationships found for aminoalkylindoles. This work provides valuable information for the design of new cannabinoid ligands, allowing us to save time and resources by directing the synthesis toward obtaining the most promising molecules. Further functional studies are required to evaluate agonism/ antagonism activity.
